# A microdeletion in the GRHL2 Gene in two unrelated patients with congenital fibrosis of the extra ocular muscles

**DOI:** 10.1186/s13104-017-2888-y

**Published:** 2017-11-06

**Authors:** Khaled K. Abu-Amero, Altaf A. Kondkar, Arif O. Khan

**Affiliations:** 1 Glaucoma Research Chair, Department of Ophthalmology, College of Medicine, King Saud University, Riyadh, Saudi Arabia; 2 Department of Ophthalmology and Visual Sciences, University of Illinois at Chicago, Chicago, IL 60612 USA; 3 Division of Pediatric Ophthalmology, King Khaled Eye Specialist Hospital, Riyadh, Saudi Arabia; 4 Eye Institute, Cleveland Clinic Abu Dhabi, Abu Dhabi, United Arab Emirates

**Keywords:** arrayCGH, CCDD, Congenital fibrosis, *GRHL2*

## Abstract

**Objective:**

Congenital fibrosis of the extraocular muscles type 1 (CFEOM1) is known to be caused by mutations in *KIF21A* or *TUBB3* or other known genes (*SALL4, CHN1, HOXA1*). However, affected children may harbor other genetic defects. Therefore, a candidate gene analysis (*KIF21A, TUBB3 SALL4, CHN1, HOXA1*) and a high-resolution array comparative genomic hybridization (arrayCGH) was performed in two unrelated children with sporadic CFEOM1.

**Results:**

Two unrelated Saudi patients did not have any mutation(s) after sequencing the full coding regions of *SALL4*, *CHN1*, *HOXA1*, and *TUBB3* genes; and exons 8, 20, and 21 of the *KIF21A* gene. However, arrayCGH revealed a 3.17 Kb deletion at chromosome 8p22 with copy number state equal to 1, indicating a heterozygous deletion. This deletion was absent in proband’s mother or father or 220 unrelated healthy individuals of similar ethnicity. The deletion encompassed only one functional gene, *GRHL2*, which encodes a transcription factor. In humans, defects in this gene are a cause of non-syndromic sensorineural deafness, autosomal dominant type 28 (DFNA28). We speculate that *GRHL2* gene may have a role in orbital innervations and the defect in this gene (deletion) may be related to the CFEOM1 phenotype in these two children.

## Introduction

Congenital cranial dysinnervation disorders (CCDDs) include most congenital, static abnormalities of ocular motility and certain additional syndromes primarily involving lid and facial muscle innervations [1]. Individual CCDDs can be sporadic (e.g., most Duane retraction syndrome) [2], autosomal dominant (e.g., congenital fibrosis of the extraocular muscles type [CFEOM] type 1 or 3 [3, 4], autosomal recessive (e.g., the *HOXA1* spectrum or CFEOM2 [5, 6], or chromosomal in origin [7]. Certain types of CCDDs affect only ocular motility (e.g., CFEOM1), however, several other types of CCDDs are now known that exhibit non-ophthalmic associations involving neurologic, neuroanatomic, cerebrovascular, cardiovascular, and skeletal abnormalities as reviewed elsewhere [8]. Studies in the past have associated syndromic CCDD with chromosomal copy number variations (CNVs) including deletion(s) [9, 10], duplication(s), translocation(s) [11] and the presence of 22 marker chromosome [12]. The methods employed in these studies had a limited resolution of 5–10 Mb for standard karyotyping, 3–5 Mb for FISH probes, or 80–200 Kb for BAC clones. In contrast, currently available high-resolution array comparative genomic hybridization (arrayCGH) has the ability to detect small and potentially symptomatic CNVs into the range of 1 Kb. Using this approach, we have identified different CNVs in several patients with syndromic Duane retraction syndrome [7, 13–15].

In this report, we describe two unrelated Saudi patients with CFEOM1, both of whom did not have detectable CCDD mutations by candidate gene analysis, but harbored a similar recurrent CNV deletion in the Grainyhead Like transcription factor 2 (*GRHL2*) gene (Gene ID: 79977).

## Main text

### Materials and methods

#### Settings and patient information

The study was conducted under an institutional review board ap-proved protocol for genetic study of eye movement disorders at the King Khalid Eye Specialist Hospital, Riyadh, Saudi Arabia (0424-P). Written, informed consents were obtained from all participating individuals. Informed consents were signed from both the proband’s parents. The patients were examined clinically and their medical records were reviewed (as detailed in Results) at the Ophthalmic Genetics Laboratory, King Abdulaziz University Hospital, King Saud University, Riyadh, Saudi Arabia from December 2014 through June 2015.

#### Sanger sequencing of *SALL4*, *CHN1*, *HOXA1*, *TUBB3 and KIF21A* genes

All the exons of *SALL4*, *CHN1*, *HOXA1* and *TUBB3* genes were sequenced according to the protocol described previously [7]. In addition, exons 8, 20, and 21, considered as mutations hotspots in *KIF21A* gene were also sequenced as previously described in details [14].

#### Array comparative genomic hybridization (arrayCGH)

The Affymetrix Cytogenetics Whole Genome 2.7 M array (Affymetrix Inc., Santa Clara, CA, USA) was utilized to look for chromosomal alterations (deletion(s) and/or duplication(s)) in the whole genome as detailed elsewhere [13, 15].

#### Analysis of arrayCGH data

Affymetrix Chromosome Analysis Suite v1.2 (ChAS) Software (Affymetrix Inc. CA, USA) was used for arrayCGH data analysis. As currently, there is no internationally accepted consensus for analyzing data generated by high resolution arrayCGH, we devised our own [13]. Accordingly, a CNV had to satisfy the following criteria’s to be considered as potentially pathogenic: (1) CNV has to be absent in the Database of Genomic Variants (DGV; http://projects.tcag.ca/variation/) among normal controls; (2) has to be absent in our own database of unrelated healthy controls of Saudi ethnicity. So far, we have analyzed 220 normal controls by arrayCGH; (3) the detected variant has to segregate with the phenotype and should not be present in unaffected or asymptomatic family members; and (4) it included an area of the genome encompassing one or more functional gene(s). The threshold for gain or loss was adjusted to 10 Kb. We used the National Center for Biotechnology Information Human Genome Assembly Build 35 as a reference.

#### Semi-Quantitative PCR for deletion confirmation

A semi-quantitative polymerase chain reaction (PCR) method was performed on the ABI 3130*xl* Genetic Analyzer (Applied Biosystems, Foster City, CA, USA) to confirm the arrayCGH findings by measuring the sizes and fluorescence peak intensities of the gene encompassing the chromosomal variation as described previously [13, 15].

## Results

### Patient clinical evaluation

**Fig. 1:**
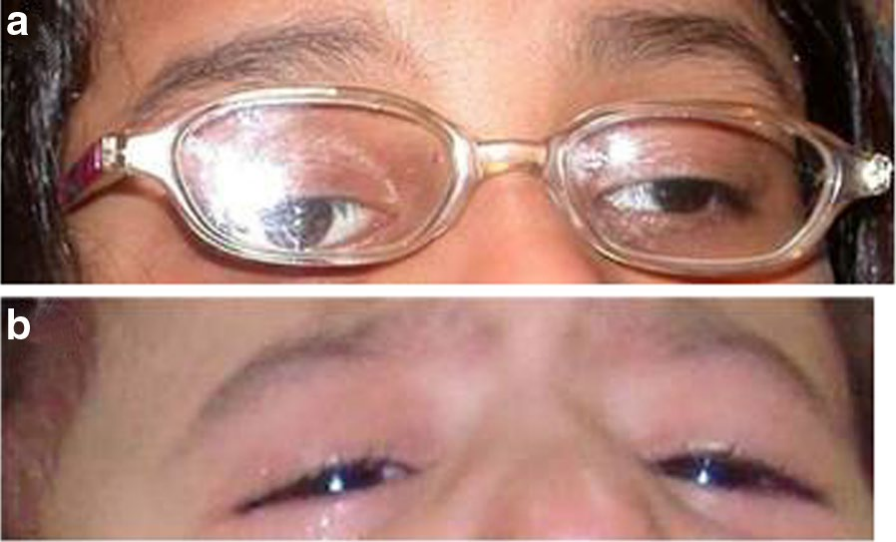
**a** (Patient 1) this 5-year old girl adopted a chinup position to compensate for her bilateral hypotropia, elevation deficiency, and ptosis. She also preferred a left face turn to fixate with her preferred right eye. With her compensatory head position there is a moderate left exotropia. **b** (Patient 2) this 22 month old boy adopted a chin-up position to compensate for his bilateral hypotropia, elevation deficiency, and ptosis. With his compensatory head position there was no horizontal strabismus

Patient 1 is a 5-year old girl and was the 5th of six siblings born to non-consanguineous parents. Pregnancy and delivery were unremarkable, and there was no family history of strabismus. The girl adopted a chinup position and left face turn for fixation with her preferred right eye (Fig. 1a). Visual acuity was 20/30 in either eye. In forced primary position there was bilateral mild hypotropia and an exo-tropia of approximately 50 prism diopters with almost complete ophthalmoplegia (only limited abduction in the right eye and limited abduction and adduction in the left eye). There was also bilateral mild true ptosis. Pupils were miotic (23 mm) but dilated well with anti-cholinergic drops. Cycloplegic refraction and fundus examination were also unremarkable. There was no evidence for decreased hearing or anterior segment abnormalities.

Patient 2 is a 22 month old boy who is the 5th child of first-cousin parents. Pregnancy and delivery were unremarkable with no family history of strabismus. The boy adopted a chinup position because of bilateral hypotropia with inability to elevate both eyes and bilateral moderate true ptosis (Fig. 1b). Extraocular motility was otherwise difficult to assess because of cooperation issues, but there was mild horizontal ophthalmology as well. There was no Bell phenomenon. There was no evidence for ocular preference. Pupils were normal. Fundus examination was grossly normal and there was no significant refractive error. Hearing was normal and there was no anterior segment abnormalities.

#### Sequencing of *SALL4*, *CHN1*, *HOXA1*, *TUBB3*, and *KIF21A*

No sequence change or mutation(s) were detected in *SALL4*, *CHN1*, *HOXA1*, and *TUBB3* upon sequencing the entire coding region of these genes. Similarly, no variations were detected after sequencing exons 8, 20, and 21, the known hot-spots of the *KIF21A* gene.

#### ArrayCGH results

We initially detected a total of 37 CNVs (14 deletions and 23 duplications in different chromosomes). Of these, 15 (40.5%) were detected in the asymptomatic father and 13 (35.1%) in the asymptomatic mother and these variants were thus eliminated from further analysis. Of the remaining 9 CNVs, 8 (88.8%) were detected in our database of full genome CNVs from 220 normal controls of similar ethnicity and were thus filtered out. The remaining CNV that deemed important was a deletion in chromosome 8 extending from 102,670,305 to 102,673,473 (size = 3.17 Kb) at the 8p22 cytogenetic band. The copy number was equal to 1, indicating that this deletion was likely to be heterozygous. This deletion was likely to be due novo and segregated with the syndrome described here because it was not detected in the proband’s mother or father or 220 unrelated healthy individuals of similar ethnicity. No other known or potentially pathological chromosomal copy number changes were detected in this patient using the criteria detailed in the method’s section. The confidence value calculated by the ChAS software was 88% with a marker count of 20 spanning the deleted area. This is a good number of markers in the deleted area and give confidence of the deletion present in this region. The deleted area on chromosome 8 was confirmed by semi-quantitative PCR as detailed in the methods. The mean (standard deviation) of three separate readings of fluorescence peak area was 653.4 (9.8) for the proband, 898 (9.5) for the mother, 917.9 (24.5) for the father, and 895.2 (16.1) for a normal male control of similar ethnicity. This 3.17 Kb deletion region encompassed only one functional gene, *GRHL2*. The protein encoded by this gene is a transcription factor that can act as a homodimer or as a heterodimer with either GRHL1 or GRHL3. Defects in this gene are one of the genetic causes of non-syndromic sensorineural deafness, autosomal dominant type 28 (DFNA28).

## Discussion

Although CFEOM1 is mainly caused by *KIF21A* or *TUBB3* mutation(s) [1], there are previous reports indicating that affected children from the Arabian Peninsula sometimes do not harbor detectable mutations in either gene [16, 17]. In two such children from two different families we report a small deletion encompassing *GRHL2* and suggest that it may have been related to the phenotype. To our knowledge, the two patients are not related and both belong to different tribes from different parts of the Kingdom.

GRHL2 is a mammalian homolog of Drosophila protein grainy head (GRH), which is part of a family of transcription factors that play a role in epithelial morphogenesis [18]. The development and cell maintenance of the vertebrate eye requires the synchronized action of number of genes including many transcription factors [19]. Mutations and deleterious changes in these genes encoding transcription factors could hamper the developmental regulatory networks and may result in general developmental defects associated with eye-related problems [20, 21]. GRHL2 knockout is embryonically lethal in mice, causing severe facial and neural tube defects [22]; and mutant zebrafish exhibit inner ear defects and abnormal swimming positions [23]. These studies clearly establish a crucial role of *GRHL2* in embryonic development, and our finding of *GRHL2* deletion in two unrelated CFEOM patients suggests a role for the gene in orbital innervation. An involvement of *GRHL2* in other physiological processes have also been described. *GRHL2* (alias *TFCP2L3*, MIM 608576) mutation has been reported in childhood-onset progressive autosomal dominant hearing loss [DFNA28 (MIM 608641)] [24]; however, there was no evidence for hearing loss in either of our patients. Homozygous mutations in this gene have been implicated in an autosomal- recessive ectodermal dysplasia syndrome [25]. Besides, other polymorphic sequence variants in *GRHL2* have also been implicated in age-related hearing impairment and noise-induced hearing loss [26, 27]. Interestingly, *GRHL2* has been shown to regulate transcription factor zinc finger enhancer binding protein 1 (ZEB1) [28]. Mutations in *ZEB1* are known to be pathogenic causing posterior polymorphous corneal dystrophy-3, late-onset Fuchs endothelial corneal dystrophy, and keratoconus [29]. However, there was no evidence for corneal disease in either of our patient. It has been shown that, *GRHL2* and *ZEB1* transcription factors form a double negative regulatory feed-back loop [28, 30]. In addition, ZEB1 transcriptionally represses genes of the *miR*-*200* family, and that *GRHL2* up-regulates the expression of miR-200b/c family members in breast cancer cells [28]. Based on our finding of *GRHL2* deletion observed in our patients with CFEOM, it is possible that the highly complex, interconnected *GRHL2*/*ZEB1*/*miR*-*200* regulatory system may be involved in orbital innervation [30].

## Limitations

Development of normal ocular motility is a complex process that is not yet fully understood. Although an in-depth genetic screening using both candidate-gene and a genome-wide approach was conducted, the conclusion of this study is based on observation in only two cases with CFEOM1. A more firm link between the *GRHL2* gene and this syndromic CCDD variant may be established in the future if these observations are replicated in more patients. Other genetic abnormalities not tested for by the techniques employed here could be responsible for this syndrome. Lastly, the role of epigenetic or environmental factors in contributing or directly causing this phenotype has not been ruled out.

## Abbreviations

ArrayCGH: array comparative genomic hybridization
CCDD: congenital cranial dysinnervation disorders
CFEOM: congenital fibrosis of the extraocular muscles type
CNV: copy number variation
DFNA28: sensorineural deafness autosomal dominant type 28
*GRHL2*: Grainyhead Like transcription factor 2
PCR: polymerase chain reaction
ZEB1: zinc finger enhancer-binding protein 1
